# Outcomes of surgical resection for pulmonary metastasis from ovarian cancer

**DOI:** 10.1186/s13019-020-01231-x

**Published:** 2020-07-23

**Authors:** Ryu Kanzaki, Jiro Okami, Koji Takami, Teruo Iwasaki, Naoki Ikeda, Yasunobu Funakoshi, Yasushi Sakamaki, Ken Kodama, Hideoki Yokouchi, Yoshihisa Kadota, Naoko Ose, Yasushi Shintani, for Thoracic Surgery Study Group of Osaka University (TSSGO)

**Affiliations:** 1 Department of General Thoracic Surgery, Osaka University Graduate School of Medicine, 2-2 (L5), Yamadaoka, Suita, 565-0871 Japan; 2 Department of General Thoracic Surgery, Osaka International Cancer Institute, Osaka, Japan; 3 Department of Thoracic Surgery, National Hospital Organization Osaka National Hospital, Osaka, Japan; 4 Department of General Thoracic Surgery, Japan Community Healthcare Organization Osaka Hospital, Osaka, Japan; 5 Department of Thoracic Surgery, Sakai City Medical Center, Sakai, Japan; 6 Department of Thoracic Surgery, Osaka General Medical Center, Osaka, Japan; 7 Department of Thoracic Surgery, Osaka Police Hospital, Osaka, Japan; 8 Department of Thoracic Surgery, Yao Municipal Hospital, Yao, Japan; 9 Department of Thoracic Surgery, Suita Municipal Hospital, Suita, Japan; 10 Department of Thoracic Surgery, Osaka Habikino Medical Center, Habikino, Japan

**Keywords:** Pulmonary metastasis, Surgery, Ovarian cancer

## Abstract

**Background:**

Due to its rarity, information on pulmonary metastasectomy for pulmonary metastasis from ovarian cancer is limited.

**Methods:**

Cases of pulmonary metastasectomy for ovarian cancer were collected in a multi-institutional setting and the outcomes were analyzed.

**Results:**

Among 1508 cases in which pulmonary resection was performed to treat pulmonary metastasis from tumors of various organs, 6 cases (0.4%) involved pulmonary metastasis from ovarian cancer. The mean age was 61 years (range, 39–75 years). The histological types were undifferentiated carcinoma in 2 patients, and clear cell adenocarcinoma, serous papillary cystadenocarcinoma, serous adenocarcinoma, and endometroid adenocarcinoma in 1 patient each. One patient (17%) had a history of liver metastasis at the time of pulmonary resection. The median disease-free interval was 22 months (range, 0 [synchronous]-188 months). The tumor was solitary in 5 patients (83%). The mean tumor size was 15 mm (range, 5–23 mm). All 6 patients underwent complete resection. The type of resection was wide wedge resection in 3 patients, segmentectomy in 2 patients, and lobectomy in 1 patient. Four patients (67%) received postoperative chemotherapy. Thus far, 4 patients (67%) have experienced recurrence after pulmonary resection. In terms of outcomes, 1 patient who had synchronous pulmonary metastasis with the primary tumor died in the early period after pulmonary resection, 1 patient is alive without recurrence after a short follow-up period (5 months), 3 patients have achieved mid- to long-term survival and are alive with disease (38–61 months), and 1 patient achieved long-term (61 months) disease-free survival.

**Conclusions:**

Patients with pulmonary metastasis from ovarian cancer who fulfill the eligibility criteria for pulmonary metastasectomy are rare. Pulmonary metastasectomy for ovarian cancer can provide favorable outcomes in highly selected patients. Patients with synchronous pulmonary metastasis from ovarian cancer are not good candidates for pulmonary metastasectomy.

## Background

Ovarian cancer is a leading cause of death in gynecologic malignancies [1]. The poor prognosis of ovarian cancer is partly explained by the following factors: more than 70% of patients present with advanced disease at the time of the diagnosis and more than 60% of patients experience recurrence after primary treatment [2]. Epithelial tumors, which account for most ovarian cancers, usually metastasize directly to the peritoneum; hematogenous metastasis is rare [3].

The mainstay of treatment for recurrent ovarian cancer is chemotherapy [4]. Surgical treatment could be also a viable treatment option for recurrent ovarian cancer; however, most reports on the topic are limited to patients with metastasis in the peritoneal cavity [2, 5]. Reports on surgical treatment for distant metastasis from ovarian cancer are limited. Kwon et al. [6] reported that surgical resection for single or symptomatic brain metastasis from ovarian cancer prolonged survival. Burton et al. [7] reported the outcomes of 20 cases of surgical treatment for recurrent ovarian cancer, including 11 patients with hepatic metastasis and 1 patient with pulmonary metastasis, and the 5-year overall survival rate was 45%.

Pulmonary metastasectomy (PM) is an established treatment for various types of cancer, including colorectal cancer and renal cell carcinoma [8]. However, due to its rarity, information on PM for pulmonary metastasis from ovarian cancer is limited. In the present study, we analyzed the outcomes of PM for ovarian cancer in a multi-institutional setting.

## Patients and methods

In this study, data from 10 different thoracic surgery departments belonging to the Thoracic Surgery Study Group of Osaka University were analyzed. From January 2006 to December 2015, 1508 patients in 10 hospitals underwent pulmonary resection to treat pulmonary metastasis originating from tumors of various organs. Among them, 6 cases (0.4%) from 4 hospitals underwent PM for pulmonary metastasis from ovarian cancer. Patients with ovarian granulosa cell tumor (GCTs) are excluded from this study because these are classified as borderline malignancies, which are considered to be a different entity from malignancy. In the present study, these 6 patients were retrospectively reviewed. The study protocol was approved by the institutional review boards of all participating hospitals, including the Ethics Committee of Osaka University Hospital (control number 17255–2). Patients who underwent pulmonary resection for the purpose of surgical biopsy alone were excluded from this study. The preoperative diagnoses of the pulmonary nodules were made based on chest computed tomography (CT) findings. In all patients, the primary tumors were pathologically diagnosed prior to pulmonary resection and the primary tumor was treated by radical resection. In all cases, the lung specimens were histologically evaluated, and metastatic ovarian cancers were diagnosed by pathologists. Clinical information was collected from the medical records in participating hospitals.

Patients generally underwent resection of pulmonary metastases after meeting the following criteria [9]: (1) complete resection of pulmonary metastasis (or metastases) was considered to be achievable; (2) the metastatic lesion(s) was limited to the lungs, or extra-pulmonary distant metastasis was already controlled or controllable if present; (3) the patient’s primary tumor was already controlled or controllable; (4) the general condition of the patient was good, and the patient’s respiratory function was sufficient to tolerate pulmonary resection.

The type of resection and surgical approach were selected according to the size and location of recurrent pulmonary metastasis. The indications for perioperative chemotherapy and the timing of chemotherapy were determined by the gynecologists in charge after considering the extent of the disease and the general condition of the patient.

Follow-up examinations generally involved chest X-ray or chest CT, a physical examination and blood analyses, and were performed every 6–12 months after the first PM.

## Results

The patient characteristics and preoperative clinical factors are shown in Table 1. The mean age was 61 years (range, 39–75 years). In all cases, the primary cancer was completely resected. Three patients underwent surgery alone for treatment of primary tumor while 3 patients underwent surgery and both preoperative and postoperative chemotherapy or postoperative chemotherapy. The histologic type was undifferentiated carcinoma in 2 patients, and clear cell adenocarcinoma, serous papillary cystadenocarcinoma, serous adenocarcinoma, and endometroid adenocarcinoma in 1 patient each. One patient (17%) had a history of liver metastasis at the time of pulmonary resection. The median disease-free interval (DFI) was 22 months (range, 0 [synchronous] - 188 months). A solitary tumor was found in 5 patients (83%) while 1 patient had 3 tumors. The mean tumor size was 15 mm (range, 5–23 mm). (FDG-PET was performed in 5 patients; the FDG uptake (maximum standardized uptake value [SUVmax]) ranged from 1.6 to 6.6.

**Table 1:** Patient characteristics and preoperative clinical factors

Pt	Age	Pathologic stage of ovarian cancer	Pathology	Treatment mode for the primary tumor	Metastatic site other than lung before pulmonary resection	Disease-free interval (months)	Laterality	Tumor number	Tumor size (mm)	FDG uptake (SUVmax)	Lymph node involvement
1	64	I	clear cell adenocarcinoma	S	none	26	unilateral	1	23	8	no
2	72	IV	serous papillary cystadenocarcinoma	S	none	188	unilateral	1	10	1.6	no
3	75	IV	serous adenocarcinoma	C + S + C	none	10	unilateral	1	17	5.9	no
4	39	III	undifferentiated carcinoma	S + C	none	31	unilateral	1	19	6.6	yes
5	44	IV	undifferentiated carcinoma	S + C	liver	0	unilateral	1	18	5.8	no
6	72	III	endometroid adenocarcinoma	S	none	18	unilateral	3	5	not done	no

*C* chemotherapy, *FDG* 2-deoxy-2- [fluorine-18] fluoro-D- glucose, *SUVmax* maximum standardized uptake value, *S* surgery

The surgical and postoperative factors are shown in Table 2. All 6 patients underwent complete resection. One patient received preoperative chemotherapy, and achieved a radiological complete response. The surgical approach was open thoracotomy in 3 patients (50%) and video-assisted thoracoscopic surgery (VATS) in 3 patients. The type of resection was wide wedge resection in 3 patients, segmentectomy in 2 patients, and lobectomy in 1 patient. Mediastinal lymph node dissection was performed in 1 patient, sampling was performed in 1 patient, while 4 patients did not undergo lymph node dissection. Hilar lymph node involvement was observed in 1 patient. Four patients (67%) underwent postoperative chemotherapy. Thus far, 4 patients (67%) have experienced recurrence after pulmonary resection. Regarding outcomes, 1 patient died in the early period (2 months) after pulmonary resection, 1 patient remains alive without recurrence at 5 months, 3 patients have achieved mid- to long-term survival and are alive with disease (38–61 months), and 1 patient has achieved long-term disease-free survival (61 months).

**Table 2:** Surgical and postoperative factors

Pt	Preoperative chemotherapy	Surgical approach	Type of resection	Lymph node dissection	Postoperative chemotherapy	Recurrence (time after PM/ site)	Outcome
1	no	open	segmentectomy	none	yes	no	5mo NED
2	yes	open	wide wedge resection	none	no	13mo/ liver, adrenal grand, peritoneum	61mo AWD
3	no	VATS	lobectomy	mediastinal	no	no	61mo NED
4	no	VATS	wide wedge resection	sampling	yes	17mo/ mediastinal LN	50mo AWD
5	no	VATS	wide wedge resection	none	yes	1mo/ liver	2mo DOD
6	no	open	segmentectomy	none	yes	31mo/ local relapse of the primary tumor	38mo AWD

*AWD* alive with disease, *DOD* died of disease, *LN* lymph node, *NED* no evidence of disease, *VATS* video-assisted thoracoscopic surgery

## Discussion

In the present study, we demonstrated the outcomes of PM in 6 patients with pulmonary metastasis from ovarian cancer. Four of 6 patients experienced recurrence after PM, and one patient achieved long-term (61 months) disease-free survival.

Pulmonary metastasis from ovarian cancer is usually accompanied by extrathoracic metastasis; thus, isolated pulmonary metastasis is extremely rare. In the typical clinical course of ovarian cancer, which is observed in 85% of patients, the disease remains confined to the peritoneal cavity and death occurs with ascites and bowel obstruction [10]. Paik et al. [11] reported the clinical outcomes of 303 epithelial ovarian cancer patients with no gross residual disease after primary debulking surgery. In their series, 88 patients experienced recurrence, and of these, only 5 patients experienced isolated hematogenous metastasis. Based on these observations, pulmonary metastasis without other distant metastasis is considered to be a rare site of recurrence after surgical treatment of the primary cancer.

Information on PM for ovarian cancer is limited. In statistics from Japan in 2016, only 69 of 8497 (0.8%) patients who underwent PM did so for ovarian cancer [12]. Clavero et al. [13] reported the outcomes of 70 patients with metastatic gynecologic cancers who underwent PM, including 2 patients with ovarian cancer. They reported that these 2 patients achieved 3-year survival. Adachi et al. [14] reported the outcomes of 23 patients with metastatic gynecologic cancers who underwent PM, including 5 patients with ovarian cancer. These 5 patients also had good outcomes, with a 5-year overall survival rate of 100%. Although these 2 reports both demonstrated favorable overall survival after PM for ovarian cancer, the details of the individual cases were not shown. A review of the relevant literature identified 3 additional patients who underwent PM for ovarian cancer (Table 3) [15–17]. In this table, borderline malignancies, including GCT are excluded. In 2 cases, the histological type was mucinous cystadenocarcinoma and cavity formation was observed. In 2 cases, a history of liver metastasis was present at the time of PM. All 3 cases had a relatively longer DFI, of more than 6 years. All the 3 cases experienced recurrence after PM. When these 3 cases are considered with our 6 cases, 7 of the 9 patients developed recurrence while only 1 achieved long-term disease-free survival. In patients who experienced recurrence after PM, the contribution of surgical resection to the outcome cannot be assessed independently because other factors, including chemotherapy could also have contributed to the outcome. Based on the clinical data obtained thus far, PM for pulmonary metastasis from ovarian cancer could only provide favorable outcomes for highly selected patients.

**Table 3:** Reported cases of pulmonary metastasectomy for ovarian cancer

Author	Year	Age	Pathologic stage of ovarian cancer	Pathology	Metastatic site other than lung before pulmonary resection	DFl	Tumor number	Tumor size (mm)	Type of resection	Recurrence (time after PM/ site)	Outcome
Kimura [15]	1995	62	III	endometroid adenocarcinoma	liver	75 months	5	N.A.	lobectomy, wide wedge resection	14mo/ pulmonary metastasis (resected)	23mo/NED
Toishi [16]	2011	70	III	mucinous cystadenocarcinoma	liver, peritoneum	154 months	1	15	lobectomy	11mo/ liver, paraaortic LN	15mo/ AWD
Kita [17]	2015	52	I	mucinous cystadenocarcinoma	no	7 years	1	25	segmentectomy	12mo/ peritneum	24mo/ DOD

*AWD* alive with disease, *DFI* disease-free interval, *DOD* died of disease, *LN* lymph node, *N.A.* not available, *NED* no evidence of disease, *PM* pulmonary metastasectomy

DFI is reported to be an important prognostic factor after PM in various types of cancers [18]. Cormio et al. [3] reported that patients with shorter DFI had a poor prognosis in patients with distant metastasis from ovarian cancer. In the present study, the DFI ranged from 0 to 188 months. In case 2, whose DFI was 188 months, the patient experienced recurrence after PM; however, the patient was alive with disease at 61 months after PM. This observation might mean that a long DFI reflects less aggressive tumor characteristics (i.e., lower proliferation rate of cancer cells). The PET-CT findings support this notion. It is reported that the SUVmax and SUVmean were moderately correlated with Ki67 index in ovarian cancer [19]. Indeed, the SUVmax of the pulmonary nodule was relatively low, 1.6 in our case 2. On the other hand, the case with synchronous pulmonary metastasis, (case 5) had a worse prognosis, experienced liver metastasis at 1 month after PM, and died 2 months after PM. In a recent report using the SEER database, pulmonary metastasis was associated with the worst overall survival among other sites of metastasis in patients with stage IV ovarian cancer [20]. Based on our experience and these findings, it is demonstrated that patients with synchronous pulmonary metastasis from ovarian cancer are not good candidates for PM. Because a longer interval between the detection of pulmonary metastasis and PM was reported not to worsen the outcomes of patients who undergo PM [21], having an optimal observation period is important for deciding the indications for PM. In case 2, we observed the patient for 2 months after the detection of the disease before pulmonary resection; however, the patient should have been observed for longer. Based on our experience, we believe that observation for at least 3 months is needed to decide the indication for PM in patients with synchronous pulmonary metastasis from ovarian cancer.

This study has certain limitations. Recently, new treatment modes such as immunotherapy and molecular targeted therapy have obtained a rapid development [22, 23]. Because we have only database for surgical cases of pulmonary metastasis from ovarian cancer, efficiency of these new treatment modes for pulmonary metastasis from ovarian cancer could not be evaluated. Validation of efficiency of PM in comparison with these new treatment modes is future task.

## Conclusions

Patients with pulmonary metastasis from ovarian cancer who fulfill the eligibility criteria for PM are rare. PM for ovarian cancer can provide favorable outcomes in highly selected patients. Patients with synchronous pulmonary metastasis from ovarian cancer are not good candidates for PM.

## Data Availability

All of the data used in the present study are preserved in Department of General Thoracic Surgery, Osaka University Graduate School of Medicine and are available from the corresponding author on reasonable request.

## Abbreviations

CT: Computed tomography
DFI: Disease-free interval
FDG-PET: Fluorine-18–2-fluoro-2-deoxy-d-glucose positron emission tomography and computed tomography
GCT: Ovarian granulosa cell tumor
PM: Pulmonary metastasectomy
SUVmax: Maximum standardized uptake value
VATS: Video-assisted thoracoscopic surgery.
